# MicroRNA-196a & microRNA-101 expression in Barrett's oesophagus in patients with medically and surgically treated gastro-oesophageal reflux

**DOI:** 10.1186/1756-0500-4-41

**Published:** 2011-02-27

**Authors:** Sebastien Haiart, David I Watson, Mary P Leong, David Astill, Tim Bright, Damian J Hussey

**Affiliations:** 1 Department of Surgery, Flinders University, Flinders Medical Centre, Room 3D211, Bedford Park, South Australia 5042, Australia; 2 Department of Anatomical Pathology, Flinders Medical Centre, Room 4D309, Bedford Park, South Australia 5042, Australia

## Abstract

**Background:**

Proton pump inhibitor (PPI) medication and surgical fundoplication are used for the control of gastro-oesophageal reflux in patients with Barrett's oesophagus, but differ in their effectiveness for both acid and bile reflux. This might impact on the inflammatory processes that are associated with progression of Barrett's oesophagus to cancer, and this may be evident in the gene expression profile and microRNA expression pattern in Barrett's oesophagus mucosa. We hypothesised that two miRNAs with inflammatory and oncogenic roles, miR-101 and miR-196a, are differentially expressed in Barrett's oesophagus epithelium in patients with reflux treated medically vs. surgically.

**Findings:**

Mucosal tissue was obtained at endoscopy from patients with Barrett's oesophagus whose reflux was controlled by proton pump inhibitor (PPI) therapy (n = 20) or by fundoplication (n = 19). RNA was extracted and the expression of miR-101 and miR-196a was measured using real-time reverse transcription - polymerase chain reaction. There were no significant differences in miR-101 and miR-196a expression in Barrett's oesophagus epithelium in patients treated by PPI vs. fundoplication (p = 0.768 and 0.211 respectively). Secondary analysis showed a correlation between miR-196a expression and Barrett's oesophagus segment length (p = 0.014).

**Conclusion:**

The method of reflux treatment did not influence the expression of miR-101 and miR-196a in Barrett's oesophagus. This data does not provide support to the hypothesis that surgical treatment of reflux better prevents cancer development in Barrett's oesophagus. The association between miR-196a expression and Barrett's oesophagus length is consistent with a tumour promoting role for miR-196a in Barrett's oesophagus.

## Background

Barrett's oesophagus is characterised by the development of a metaplastic columnar mucosa within the distal oesophagus. This develops as a response to gastro-oesophageal reflux. Its clinical significance is its potential to become dysplastic, and then to progress in some individuals to oesophageal adenocarcinoma. Currently, Barrett's oesophagus is the only identifiable precursor lesion for oesophageal adenocarcinoma [1].

Treatment of gastro-oesophageal reflux in patients with Barrett's oesophagus is thought to be important, as oncogenic progression may be associated with inflammation, and inflammation can be minimised by effective control of reflux [2]. Reflux control is achieved either by medication (usually proton-pump inhibitors - PPI's) or surgery (fundoplication). Although both effectively relieve reflux symptoms, only surgery constructs a physical barrier which prevents gastric contents actually entering the oesophagus. By contrast, medication reduces gastric acid production, and thereby changes the composition of the refluxate. This means that some reflux continues to occur, even though symptoms can be well controlled. Also, because the duration of effect of medication can be less than 24 hours [3], medical treatment can be associated with breakthrough acid and/or bile reflux, and this can have anti-apoptotic, proliferative and genotoxic effects in Barrett's oesophagus epithelium [4,5]. Hence, it is can be hypothesised that the distal oesophageal epithelium in patients with PPI treated Barrett's oesophagus may be more prone to ongoing inflammatory oncogenic stimuli, and this may be evident in the gene expression profile exhibited in the Barrett's oesophagus epithelium. Supporting this, it has recently been reported that levels of pro-inflammatory cytokines, including IL-1β, IL-1α, and IL-8, are elevated in Barrett's oesophagus epithelium in patients treated with PPI's, compared with patients treated by fundoplication [6].

MicroRNAs (miRNAs) are 21-22 nucleotide long, non-coding RNA segments, that regulate gene expression by binding to complementary sequences in the 3'UTR of target mRNAs, thereby interfering with mRNA translation [7]. Bioinformatic predictions estimate that a single miRNA can regulate the expression of hundreds of genes [8]. It is possible that miRNAs associated with oncogenic and inflammatory processes may be differentially expressed in Barrett's oesophagus epithelium from individuals who have medically versus surgically treated gastro-oesophageal reflux disease. To explore this possibility, we evaluated two miRNAs which are known to be associated with oncogenic processes, miR-196a and miR-101 [9-12], and we compared their expression in Barrett's oesophagus epithelium in patients with medically versus surgically treated reflux.

## Methods and materials

### Patient selection

Tissue samples for this study were obtained at endoscopy from individuals who had previously been recruited into two randomised trials of Barrett's oesophagus ablation techniques, conducted within our institution [13,14]. Entry criteria for the trials entailed: age 18-75 years, endoscopy appearances consistent with Barrett's oesophagus, histopathology confirmation of metaplastic columnar mucosa with features of intestinal metaplasia, reflux symptoms fully controlled by either regular high dose PPI medication, or surgery (fundoplication). Exclusion criteria were: no evidence of Barrett's oesophagus, ulcerative oesophagitis at endoscopy, uncontrolled reflux symptoms, high-grade dysplasia in Barrett's oesophagus, and oesophageal cancer. Full details of the recruitment of individuals has been reported elsewhere [13,14]. For the current study, endoscopy derived oesophageal mucosal tissue biopsies were obtained from individuals with Barrett's oesophagus at the time of entry into the clinical trials, before any ablative therapy had been undertaken. Tissue samples were obtained from 20 individuals who had gastro-oesophageal reflux controlled by PPI, and from 19 individuals who had undergone a previous surgical fundoplication for the control of reflux. Of the patients who had undergone surgery for reflux 10 had undergone a Nissen (360°) fundoplication, 1 a posterior partial fundoplication, and 8 an anterior partial fundoplication. In all patients, reflux symptoms were fully controlled at entry into the study according to the following criteria: (1) no reflux symptoms, and an intact fundoplication at endoscopy or (2) no reflux symptoms, consuming regular PPI medication and no oesophagitis at endoscopy. All individuals gave written informed consent for participation in this study. The study protocol was approved by the Flinders Clinical Research Ethics Committee.

### Tissue collection

Endoscopic biopsy samples were obtained according to a modified Seattle Protocol; using endoscopic jumbo forceps, commencing 1 cm above the gastro-oesophageal junction, and then continuing every 2 cm proximally along the length of the Barrett's oesophagus. At each level, 4 quadrant biopsies were obtained for conventional histopathology analysis. An additional 2-3 endoscopic mucosal biopsies were obtained for molecular biology studies (this study), and they were immediately stored in RNAlater (Ambion) and frozen at -20°C until required. For this study we used the RNAlater collected biopsy samples collected from the most distal level of endoscopically visualised Barrett's oesophagus epithelium, which was confirmed by histopathology to be from columnar mucosa with intestinal metaplasia. These samples were collected approximately 1 cm above the gastroesophageal junction.

### Biopsy processing, nucleic acid extraction, and quantitative reverse transcription-polymerase chain reaction (qRT-PCR) analysis

Selected endoscopic biopsies were thawed in RNAlater as required. 30% of each sample was then dissected from the thawed tissue sample, fixed in formalin, embedded in paraffin, and then processed for conventional histopathology. This step was used to confirm that the biopsy contained only columnar epithelium with intestinal metaplasia. The methods for this have described elsewhere [15]. The remaining 70% of the thawed biopsy had any remaining RNAlater removed, and was then processed in Trizol (Invitrogen). Details of the RNA extraction procedure and qRT-PCR method have been described fully in a previous report [16]. The following Applied Biosystems assays were used for TaqMan^® ^PCR: miR-101, no. 002253; miR-196a, no. 000495; RNU44, no.001094. MiR-101 and miR-196a expression was then determined, and was normalised with small nuclear RNA, RNU44 expression. Quantitative analysis was performed using Q-Gene software [17].

### Statistical Analysis

miRNA levels were extracted from the RotorGene software and transferred into Microsoft Excel. The Mann-Whitney U test was used to assess differences between expression levels. Spearman's correlation coefficient was used to evaluate correlations between miRNA expression and the length of the Barrett's oesophagus segment. P < 0.05 was determined to be statistically significant.

## Results

Demographic and Barrett's oesophagus associated parameters are summarised in Table 1. There were no significant differences in the gender ratio and Barrett's oesophagus segment length between the groups of patients whose reflux was treated medically vs. surgically. However, there was a significant difference in the age of patients for the 2 study groups (p = 0.0039). Table 2 summarises the expression of miR-101 and miR-196a for the two treatment groups. The treatment of gastro-oesophageal reflux (medical vs surgical) did not reveal differential expression of either miRNA.

**Table 1:** Age, sex and Barrett's esophagus segment length in patients with medically vs. surgically treated gastro-esophageal reflux

	Surgical (n = 19)	Medical (n = 20)	p value
Age (yrs)	50.0 (44.4, 54.6)	59.5 (55.4, 61.8)	0.0039

Gender (M:F)	16:3	15:5	0.6948

Barrett's esophagus segment length (cm)	3.0 (2.5, 5.4)	3.0 (2.4, 4.1)	0.71

All figures are median with 95% confidence intervals. P values are derived from Mann-Whitney U test (Age and Barrett's esophagus segment length), or Fisher's exact test (Gender).

**Table 2:** Relative expression levels for miRNAs in patients with Barrett's oesophagus with medically vs. surgically treated gastro-esophageal reflux

	Surgical (n = 19)	Medical (n = 20)	p value
**miR-101**	0.0080 (0.0071, 0.0145)	0.0102 (0.0073, 0.0126)	p = 0.768

**miR-196a**	0.0017 (0.0011, 0.0032)	0.0022 (0.0018, 0.0054)	p = 0.211

All figures are median of normalised relative expression (95% confidence intervals).

The p value is derived from a Mann-Whitney U test

Further data analysis showed an association between miR-196a expression levels and the length of the Barrett's oesophagus segment, but no association between miR-101 and length. Table 3 summarises expression in patients with Barrett's oesophagus segment lengths of less than or equal to 3 cm versus greater than 3 cm. MiR-196a levels were significantly higher in individuals with a Barrett' segment length of more than 3 cm. We also identified a significant correlation between the length of the Barrett's oesophagus segment and the expression of miR-196a (rho = 0.389, p = 0.014, Spearman's correlation coefficient), but not miR-101 (rho = -0.183, p = 0.308).

**Table 3:** Association between miR-196a and miR-101 expression vs. Barrett's esophagus segment length

	Barrett's ≤ 3 cm	Barrett's > 3 cm	p value
**miR-101**	0.0099 (0.0073, 0.0130)	0.0082 (0.0069, 0.0144)	p = 0.920

**miR-196a**	0.0010 (0.0009, 0.0028)	0.0033 (0.0022, 0.0064)	p = 0.014

All figures are median of normalised relative expression (95% confidence intervals). The p value is derived from a Mann-Whitney U test.

## Discussion

Barrett's oesophagus is the only known risk factor for the development of oesophageal adenocarcinoma, a cancer which has been rapidly increasing in incidence, particularly in male Caucasian populations [1]. It develops following long standing gastro-oesophageal reflux, probably as a consequence of the reflux of both gastric acid and duodenal content, the latter including bile acids and other substances which are toxic to the normal oesophageal mucosa [5].

Currently, medical treatment with PPIs is the first line treatment for gastro-oesophageal reflux in patients with Barrett's oesophagus. PPIs block gastric acid production, and thereby reduce the exposure of the oesophagus to low pH. This reduces or eliminates symptoms of gastro-oesophageal reflux. Unfortunately, PPIs can have a duration of effect of less than 24 hours, and this can mean that PPIs are not fully effective in patients with severe reflux [3]. As a consequence, higher dose or twice daily PPI therapy is often required in patients with Barrett's oesophagus. However, even this does not eliminate reflux in all patients, and some continue to reflux gastric and duodenal content into the oesophagus. Ongoing reflux of duodenal fluids which are rich in bile salts can induce an inflammatory process in the oesophageal mucosa, and bile salts substance are more toxic to cells at a neutral pH [18]. The alternative treatment for reflux is surgical. Wrapping the fundus of the stomach around the distal oesophagus (fundoplication) creates a new valve which physically prevents reflux. Fundoplication for reflux has been shown to achieve excellent control of reflux symptoms in approximately 90% of individuals at 10 years follow-up [19]. Because a fundoplication creates a new valve, preventing both acid and bile exposure in the oesophagus surgery reduces exposure of the oesophageal mucosa to bile acids, and this might prevent cancer development in Barrett's oesophagus. Circumstantial evidence supporting this includes studies which show that adenocarcinoma only occurs in the first 1 to 2 years follow up following fundoplication, but not at late follow up [20]. It is suggested that only small undetectable cancerous lesions, present before surgery, actually progress to cancer after fundoplication. Other studies, however, have shown late cancer development after fundoplication [21,22]. Irrespective of whether one believes that antireflux surgery prevents cancer or not, there is a consensus that reflux control should be maximised in patients with Barrett's oesophagus, and that this is important for cancer prevention.

In our study we selected 2 miRNAs for study in the context of medically vs surgical treated gastro-oesophageal reflux. MiRNA expression has been shown to be associated with the development of many cancers, and the expression of appropriately selected miRNAs might inform the debate about medical vs surgical treatment of reflux in patients with Barrett's oesophagus, and the potential for reflux control to impact on the progression of Barrett's oesophagus mucosa to cancer.

It is well understood that COX-2 is an inflammation-associated protein. It is positively regulated by IL-1β in Barrett's oesophagus [23], and its expression increases along the metaplasia - dysplasia - adenocarcinoma pathway [24]. COX-2 levels are induced by gastro-oesophageal reflux, and this is associated with increased proliferation and resistance to apoptosis in Barrett's oesophagus mucosa [4]. Importantly, miR-101 directly targets COX-2, and decreased miR-101 expression has been linked to apoptosis resistance and increased cell growth in the development of some cancers [11,12]. Whilst miR-101 has not been studied in the context of inflammation or oncogenic predisposition in Barrett's oesophagus before, we chose to study it in this context because IL-1β levels are elevated in Barrett's oesophagus mucosa from patients with medically vs surgically treated reflux [6], IL-1β positively regulates COX-2 [23], and COX-2 is negatively regulated by miR-101 [11,12]. Hence, we hypothesised that a difference in miR-101 levels might be observed in Barrett's oesophagus mucosa from patients with medical and surgically treated gastro-oesophageal reflux.

We selected miR-196a because a recent study by Maru et al. provided evidence that increased levels of miR-196a are associated with the progression of Barrett's oesophagus to adenocarcinoma [10]. Maru et al. observed a step-wise progression in miR-196a expression along the metaplasia - dysplasia - adenocarcinoma pathway. In another study they demonstrated that miR-196a has growth promoting and anti-apoptotic properties in oesophageal adenocarcinoma cell lines, and that it directly targets anti-inflammatory Annexin 1 [9]. Because of its demonstrated oncogenic and pro-inflammatory properties, we considered miR-196a to be a suitable candidate for our study.

In our study we observed no differences in miR-101 or miR-196a expression in Barrett's oesophagus epithelium between patients with medically versus surgically treated gastro-oesophageal reflux. Hence, our study failed to support the proposal that surgical control of reflux alters miRNA expression to reduce the risk of Barrett's oesophagus progressing to oesophageal adenocarcinoma. However, the results of our study should also not be interpreted as showing that the method of reflux control in Barrett's oesophagus does not influence cancer progression! An expanded range of biomarkers might demonstrate different results. It is also possible that miR-196a and miR-101 expression may not be sensitive indicators of inflammatory and precancerous processes in Barrett's oesophagus epithelium, even though bioinformatic predictions support a role for these miRNAs in targeting IL-1β, IL-1α, and IL-8 [25] (see miRecords at http://mirecords.biolead.org/prediction_query.php). Further, even if miR-196a and miR-101 do regulate IL-1β, IL-1α, and IL-8 in Barrett's epithelium, it is still possible that these pro-inflammatory cytokines are present at different levels in the two treatment groups in the absence of differential miR-101 or miR-196a expression. This is because their regulation may be independent of miRNAs in these therapeutic scenarios, or dependent on other miRNAs which we did not assess. As described above, IL-1β positively regulates COX-2, a direct target of miR-101. In our study we did not assess the expression of IL-1β or COX-2, although it would be interesting to determine in future studies whether the expression of one or both of these markers are inversely correlated with miR-101 expression in Barrett's oesophagus epithelium.

Because the length of the Barrett's oesophagus segment has been shown to correlate with the severity of gastro-oesophageal reflux [26-28], as well as the risk of progression to oesophageal adenocarcinoma [29], we tested a secondary hypothesis that there might be an association between miR-101 and/or miR-196a expression and the length of the Barrett's oesophagus segment. We found a positive correlation between miR-196a expression and the length of Barrett's oesophagus. Although Barrett's oesophagus length has been correlated with DNA methylation in gene promoters [30], to our knowledge only one study has assessed associations between gene expression and Barrett's oesophagus length, and no associations were observed [31]. Indirectly, our observation is in agreement with the results of Maru et al. which support the oncogenic potential of miR-196a in Barrett's oesophagus, and observations of an association between Barrett's oesophagus segment length and increased cancer risk [29]. If the length of Barrett's oesophagus correlates with the risk of cancer development, our results raise the possibility that miR-196a expression might be used as a biomarker for stratification of cancer risk in patients with Barrett's oesophagus. This possibility will need to be evaluated in further studies. Such studies might entail comparing the expression of miR-196a in Barrett's oesophagus from patients who have progressed to cancer vs. those that have not. This would require either longitudinal collection of tissues from a Barrett's oesophagus surveillance cohort, or retrospective collection of old paraffin embedded surveillance biopsy material from individuals who have progressed from Barrett's oesophagus to cancer *vs *those without cancer. The sensitivity and specificity for miR-196a expression levels vs cancer progression could be determined in this way.

There are some limitations to our study, and the data should be interpreted carefully. Firstly, there was a significant difference in age between the two reflux treatment groups, and we cannot eliminate the possibility that this might have influenced outcomes study outcomes. We hypothesise that this difference was due to a selection bias towards different treatments based on age. Secondly, we did not include any reference control tissues, so we cannot exclude the possibility that medical and surgical therapy affect miR-196a and miR-101 expression to a similar extent. Squamous epithelium from more proximally in the oesophagus could easily be obtained, but it is not and appropriate control for this study, as miRNA expression is tissue specific [10] and our study was focussed on Barrett's oesophagus mucosa. The most appropriate control tissue would be Barrett's oesophagus mucosa from patients with uncontrolled reflux, i.e. patients who had not undergone antireflux surgery who were also not using antireflux medication. Finally, we only assessed 2 miRNA biomarkers, and a different methodology would be required to more comprehensively test our original hypothesis. This could be undertaken initially as a microarray based discovery study, followed by PCR based validation of relevant miRNA markers shown to differ in Barrett's oesophagus mucosa from patients with medically vs. surgically treated reflux. Further, 24 hour ambulatory pH data might better inform the quality of reflux control and its potential impact on miRNA expression. Nevertheless, all of the patients in our study had clinically effective reflux control, and endoscopy findings which supported the clinical assessment, suggesting that the study groups were representative of patients whose reflux was managed by either effective PPI therapy or an effective fundoplication.

In summary, we found no evidence for differential expression of the oncogenic and inflammatory related miRNAs - miR-101 and miR-196a, in Barrett's oesophagus epithelium from medically versus surgically treated patients. Further studies, including microarray based analyses, are warranted to determine if any microRNAs are differentially expressed in Barrett's oesophagus mucosa in patients with gastro-oesophageal reflux treated with either PPI's or surgical fundoplication. We found a novel positive association between the expression of miR-196a and the length of the Barrett's oesophagus segment, consistent with a hypothesised tumour promoting role for miR-196a in Barrett's oesophagus.

## Competing interests statement

The authors have no competing interests to disclose.

## Authors' contributions

SH and MPL: processing and extraction of nucleic acids from tissues and qRT-PCR; DIW and TB: collection of samples; DA histopathological confirmation of tissues; DJH: supervision of the study. SH, DIW and DJH had lead roles in manuscript preparation and all authors read and approved the final manuscript.
